# Limited Density of an Antigen Presented by RMA-S Cells Requires B7-1/CD28 Signaling to Enhance T-Cell Immunity at the Effector Phase

**DOI:** 10.1371/journal.pone.0108192

**Published:** 2014-11-10

**Authors:** Xiao-Lin Li, Marjolein Sluijter, Elien M. Doorduijn, Shubha P. Kale, Harris McFerrin, Yong-Yu Liu, Yan Li, Madhusoodanan Mottamal, Xin Yao, Fengkun Du, Baihan Gu, Kim Hoang, Yen H. Nguyen, Nichelle Taylor, Chelsea R. Stephens, Thorbald van Hall, Qian-Jin Zhang

**Affiliations:** 1 Department of Biology, Xavier University of Louisiana, New Orleans, Louisiana, United States of America; 2 Clinical Oncology, K1-P, Leiden University Medical Center, Leiden, the Netherlands; 3 Department of Basic Pharmaceutical Sciences, University of Louisiana at Monroe, Monroe, Louisiana, United States of America; 4 College of Chemistry & Environmental Science, Hebei University, Hebei Province, Baoding, China; Ohio State University, United States of America

## Abstract

The association of B7-1/CD28 between antigen presenting cells (APCs) and T-cells provides a second signal to proliferate and activate T-cell immunity at the induction phase. Many reports indicate that tumor cells transfected with B7-1 induced augmented antitumor immunity at the induction phase by mimicking APC function; however, the function of B7-1 on antitumor immunity at the effector phase is unknown. Here, we report direct evidence of enhanced T-cell antitumor immunity at the effector phase by the B7-1 molecule. Our experiments *in vivo* and *in vitro* indicated that reactivity of antigen-specific monoclonal and polyclonal T-cell effectors against a Lass5 epitope presented by RMA-S cells is increased when the cells expressed B7-1. Use of either anti-B7-1 or anti-CD28 antibodies to block the B7-1/CD28 association reduced reactivity of the T effectors against B7-1 positive RMA-S cells. Transfection of Lass5 cDNA into or pulse of Lass5 peptide onto B7-1 positive RMA-S cells overcomes the requirement of the B7-1/CD28 signal for T effector response. To our knowledge, the data offers, for the first time, strong evidence that supports the requirement of B7-1/CD28 secondary signal at the effector phase of antitumor T-cell immunity being dependent on the density of an antigenic peptide.

## Introduction

It is well established that in the induction phase of CD8^+^ T-cell responses, T cells require two signals through cell-cell interactions with antigen presenting cells (APCs) for their activation and proliferation [1], [2]. Major Histocompatibility Complex class I (MHC-I) presentation of antigen to the T-Cell Receptor (TCR) serves as the first signal, while association of B7-1 (or CD80) with the CD28 molecule expressed on T cells triggers the second signal. B7-1 is not expressed on most tumor cells; therefore, if tumors express MHC-I and trigger the first signal, they may not fully activate anti-tumor specific T cells [3]; however, transfecting the B7-1 gene into tumor cells can render them capable of effectively stimulating antitumor T-cell activation, leading to cancer eradication *in vivo* [4]–[8]. The augmented antitumor T-cell responses by B7-1 expressing tumor cells occur in the induction phase of immunity.

Transporter associated with antigen processing (TAP)-deficient tumors represent immune-escape variants [9]. Presentation of MHC-I-restricted antigen in these tumors is insufficient; therefore, the induction of the T-cell responses is either difficult [10] or less efficient [11]. Introduction of the B7-1 gene into TAP-deficient tumor cells stimulates immune system to generate stronger T-cell mediated immune responses against B7-1 negative parental counterparts [10]–[12], suggesting that the induction phase of T-cell immunity is augmented by B7-1. Recent evidence indicates that CD8^+^ T cells generated by B7-1 expressing tumor cells recognized a panel of the TAP independent antigens [13]. One of the antigens, Lass5, derived from the ceramide synthase Lass5 (or Trh4/CerS5) protein, located in the endoplasmic reticulum (ER) lumen, associates with H-2D^b^ and is presented by many TAP-deficient, but not TAP-proficient, mouse cells [11], [13]. Although both TAP-proficient and TAP-deficient mouse cells express Lass5 protein, peptide/D^b^ complexes are selectively presented on TAP-deficient counterparts, most likely due to competition of TAP-mediated peptide antigens [14].

In this study, we have addressed whether expression of B7-1 on TAP-deficient tumor cells can functionally enhance T-cell immunities at the effector phase. We have confirmed that B7-1/CD28 signaling at the effector phase of immunity is required to enhance T-cell based immune response against Lass5 antigen expressed by TAP-deficient tumor cells, and this requirement can be overcome when the targets express high levels of the Lass5 antigen.

## Materials and Methods

### Ethics Statement

The Xavier University of Louisiana Institutional Animal Care and Use Committee (IACUC) approved animal protocol (012711-001BI) used in this study. C57BL/6 mice (6-week-old females) were purchased from Charles River Laboratories and were maintained in pathogen-free animal facilities at Xavier University of Louisiana. Each ventilated and sealed cage contained 5 mice with bedding materials of aspen shavings or shreds. All mice were treated in accordance with the Institute of Laboratory Animal Research (NIH, Bethesda, MD) Guide for the Care and Use of Laboratory Animals. In *in vivo* experiments, the tumor size reached a volume 30×10^2^ (mm^3^) or the mice were sacrificed by CO_2_ upon observed distress.

### Peptide

H-2D^b^ restricted peptide Lass5 (MCLRMTAVM) at 98% purification was purchased from GL Biochem Ltd (Shanghai, China) and used for this study. The peptide was dissolved in pure DMSO at a stock concentration of 10 mg/ml and stored at −20°C.

### Cell Lines and Cell Culture

Mouse TAP2-deficient RMA-S cells were transfected with either pUB6-vector or pUB6-based B7-1 cDNA [11]. The transfectants were designated as RMA-S/pUB and RMA-S/B7-1 cells and were maintained in RPMI 1640 (Mediatech Inc., Manassas, VA., USA) supplemented with 10% FCS, 2 mM L-glutamine, 100 IU/ml penicillin, 100 microgram/ml streptomycin and 20 mM HEPES and supplemented with 10 microgram/ml Blasticidin. In addition, both cell lines were further transfected with Lass5 (Trh4/CerS5) expressing LZRS-retroviral vector [14]. The Lass5-vector transfectants were designated as RMA-S/B7-1.Trh4 and RMA-S/pUB.Trh4 cells respectively.

### Hybridoma

Hybridoma producing anti-mouse NK1.1 monoclonal antibody (mAb), clone PK 136 was obtained from ATCC (Manassas, VA). Culture of the hybridoma and purification of the NK1.1 mAb was performed using a published protocol [15] with slight modification. The mAb was concentrated and purified using the ammonium sulfate method and purified mAb was obtained at a concentration of about 100 mg per milliliter and used for *in vivo* depletion of mouse NK cells.

### FACS Assays

FACS assays were performed to detect B7-1 on transfected cells and to detect the NK1.1 cell population in mouse splenocytes. B7-1 expressed on RMA-S/pUB and RMA-s/B7-1 transfectants was labeled with a FITC-conjugated anti-mouse CD80 mAb (clone 16-10A1, Biolegend, San Diego, CA, USA). The NK cell population was detected in mouse splenocytes by labeling with anti-mouse CD16/32 (Fc-receptor) mAb (clone 93, Biolegend, San Diego, CA, USA), followed by labeling with FITC-conjugated anti-mouse NK1.1 mAb (clone PK136, Biolegend, San Diego, CA, USA). After extensively washing, the cell pellets were suspended in PBS at 1×10^6^ cells/ml concentration. Expression of cell surface B7-1 molecule and NK1.1 protein was determined by using a BD FACScalibur.

### Quantitative PCR analysis of Lass5 expressing transfectants

Total RNA isolation and cDNA preparation from RMA-S/B7-1.Trh4 and RMA-S Trh4/pUB cells were performed using an RNeasy Mini Kit (Qiagen, MD, USA). Five hundred nanograms of purified total RNA were used to synthesize cDNA using a High Capacity RNA-to-cDNA Kit (Applied Biosystems, Foster City, USA). Quantitative PCR on short and long transcripts of Trh4 was done as described previously [13]. SensiMix SYBR No-ROX kit from GC Biotech Bioline (Alphen aan den Rijn, NL) was used in a C1000 Thermal Cycler (Bio-Rad, Hercules, CA, USA) and results were analyzed using Bio-Rad CFX manager software. Long Trh4 (Lass5) transcripts were amplified with Power SYBR Green Master Mix (Applied Biosystems) on a GeneAmp 7300 System (Applied Biosystems).

### Generation of Cytolytic T Lymphocytes (CTL) and ^51^Cr-release Assays

Antigens used for CTL generation were prepared using the following procedures: RMA-S/B7-1 or RMA-S/pUB cells were incubated at 26°C overnight with 100 micromole D^b^-restricted and TAP-independent Lass5 peptide [13]. Afterwards, the cells were treated with 30 microgram/ml mitomycin-c for 3-hours at 26°C and washed extensively. The peptide-pulsed RMA-S/B7-1 or RMA-S/pUB cells were then injected i.p. into C57BL/6 mice (5×10^6^ cells/mouse). After a 9-day immunization, the RMA-S/pUB- or RMA-S/B7-1-immunized mice were killed by CO_2_. The immunized spleens were re-stimulated with mitomycin-c treated, 100 micromole Lass5-pulsed RMA-S/pUB or RMA-S/B7-1 cells (1×10^7^ cells/1×10^8^ splenocytes). ^51^Cr-release assays were conducted by using target cells indicated in each figures. Percentage data were converted to logarithmic data before statistical analysis. Two-way ANOVA followed by Dunnett’s Multiple Comparison test or Unpaired Student’s t-test were performed. Results were considered significant if P value≤0.05.

### T-cell activation assays

Lass5-specific T cell clone LnB5 was generated as previously described [13]. T-cell activities were measured by intracellular IFN-gamma staining of T-cells conducted as previously described [16], [17]. In brief, 8×10^3^ Lass5-specific LnB5 cells were incubated with indicated amounts of stimulator cells for 4-h in the presence of 1 microgram/ml GolgiPlug (BD Biosciences). After incubation the cells were fixed, permeabilized and stained with PE-conjugated IFN-gamma-specific mAb, using an intracellular cytokine staining starter kit (BD Biosciences). Afterwards, the cells were stained with FITC-conjugated anti-mouse CD8a mAb and washed extensively. The cell samples were then analyzed using a FACS Calibur flow cytometer (BD Biosciences). Percentage data were converted to logarithmic data before statistical analysis. Two-way ANOVA followed by Dunnett’s Multiple Comparison test or Student’s t-test were performed. Results were considered significant if P value≤0.05.

### Reduction of CTL Killing Activity by Blocking of B7-1/CD28 Binding

mAbs against mouse B7-1 (Clone 16-10A1; Armenian Hamster IgG), CD28 (Clone 37.51; Golden Syrian Hamster IgG), and relevant purified Hamster IgG-isotype controls were purchased (eBioscience, San Diego, CA). Both mAbs were reported to functionally block B7-1/CD28 binding [18], [19]. Before adding bulk-cultured CTLs or the LnB5 T-cell clone into target cell cultures for ^51^Cr- release assays or intracellular IFN-gamma secretion assays, either T cells or target RMA-S/B7-1-culture was added with 10 microgram/ml relevant mAbs against either mouse CD28 (for CTL-culture) or mouse B7-1 (for RMA-S/B7-1-culture) for 1 hour at room temperature. The relevant purified Hamster IgG-isotype control antibody was used as an experimental control. The antibody-containing cultures were then used for ^51^Cr-release assays (for bulk-cultured CTLs) or intracellular IFN-gamma secretion assays (for LnB5 T-cells).

### *In Vivo* Tumor Growth

C57BL/6 mice were treated with three alternate procedures before tumor cell challenge. 1) The mice were immunized i.p with PBS; 2) The mice were immunized i.p. with Lass5-peptide-pulsed and mitomycin-c-treated RMA-S/pUB cells or RMA-S/B7-1 cells at 5×10^6^ cells/mouse; and 3) After one week of immunization with 5×10^6^ cells/mouse Lass5-peptide-pulsed and mitomycin-c-treated RMA-S/pUB cells, the mice were depleted of NK effectors by using concentrated NK1.1 mAb (clone 16-10A1, 0.5 mg/mouse injection). The mAb treatment was performed every other day for the first one and half weeks and once a week for the following weeks. Twenty three days post-immunization, the mice were challenged s.c. with 5×10^6^ live RMA-S/pUB or RMA-S/B7-1 cells per mouse. Tumor growth was initially detected by palpation daily, and once tumor were palpable, tumor volume was measured by a caliper and calculated by the formula V = 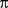 x abc/6 (where a, b, and c are the orthogonal diameters). The experimental mice were terminated at animal facility by CO2 inhalation when the tumor size reached a volume 30×10^2^ (mm^3^). Each experimental group contained 4 to 5 mice described in table 1.

**Table 1:** C57/BL6 mice used in each different experimental group.

number of mice	RMA-S/pUB -challenge*	RMA-S/B7-1-challenge*
RMA-S/pUB-immunized	4	5
RMA-S/B7-1-immunized	4	5
PBS-immunized	4	4
*NK depletion and RMA-S/pUB-immunized*	4	4

*indicates the number of mice per group.

Results of statistical analysis for mouse tumor sizes at specific time points were obtained using Paired Student *t* test, and differences were considered significant at P<0.05.

## Results

### Inhibition of RMA-S/B7-1 cell growth in immunized syngeneic mice

**Figure 1:**
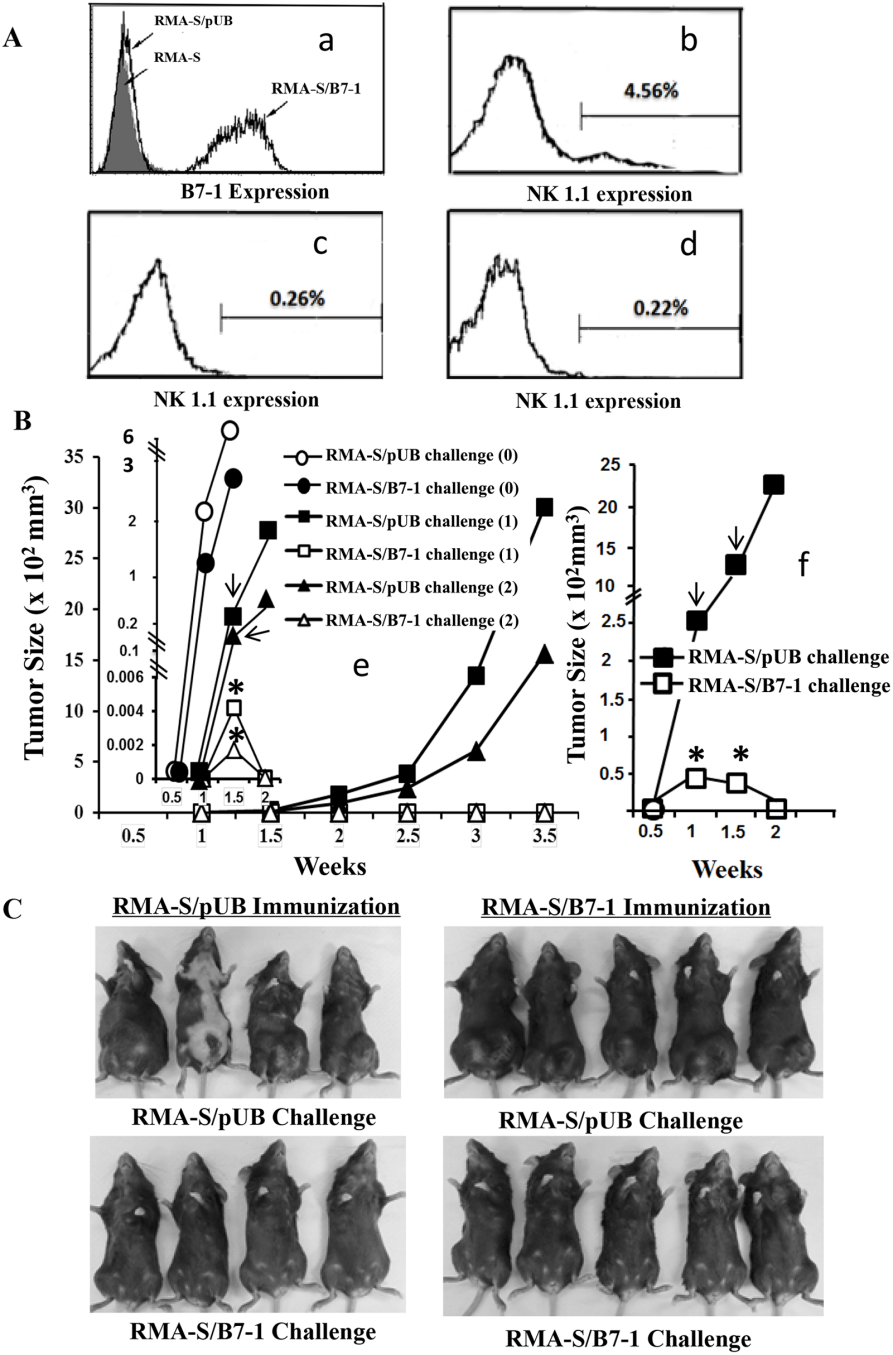
Inhibition of B7-1 expressing RMA-S tumor growth in Lass5-antigen immunized mice. A: a) B7-1 expression in the transfectants. B7-1 expression was determined by FACS assay using FITC-conjugated anti-mouse CD80 mAb; b, c and d) NK1.1 population in mouse splenocytes were detected by anti-NK1.1 mAb. b) Normal mouse splenocytes, c) and d) the splenocytes from tumor-immunized and anti-NK1.1 mAb treated mouse (c: on the tumor cell challenge time and d: end of experiment). B and C: *In vivo* tumor growth assays. B: e) mice immunized with PBS (0), Lass5-peptide-pulsed and mitomycin-c-treated RMA-S/pUB (1) or RMA-S/B7-1 (2) cells. After immunization, the mice were challenged s.c with RMA-S/pUB or RMA-S/B7-1 cells. The insert indicates tumor growth during the time point of the initial tumor cell injection through two weeks. f) Mice immunized with Lass5-peptide-pulsed and mitomycin-c-treated RMA-S/pUB cells and followed by anti-NK1.1 mAb treatment. Afterwards, the mice were challenged s.c with RMA-S/pUB or RMA-S/B7-1 cells. Statistical analysis of tumor sizes indicated significant differences between RMA-S/pUB ‘
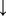
’ and RMA-S/B7-1 ‘*’ cell challenge groups at relevant time points (P value≤0.05 or 0.01). C: Tumor sizes at the endpoint were shown in the mice immunized with Lass5-peptide-pulsed and mitomycin-c-treated RMA-S/pUB or RMA-S/B7-1 cells and followed by challenge with live RMA-S/pUB or RMA-S/B7-1 cells.

B7-1 molecule expression on tumor cells can elicit anti-tumor immunity at the induction phase [11], [12], [20], [21]; however, there has been no direct evidence to support the enhancement of anti-tumor immunity at the effector phase by B7-1. To test this possibility, RMA-S cells were transfected with the B7-1 gene (designated as RMA-S/B7-1) or a relevant vector (designated as RMA-S/pUB). B7-1 expression on RMA-S/B7-1 but not RMA-S/pUB cells was confirmed by FACS assay (Fig. 1A-a).

To test if B7-1 enhanced T-cell based antitumor immunity at the effector phase, we conducted an *in vivo* tumor-growth inhibition experiment. Since RMA–S cells present a well-known H-2D^b^-restricted Lass5 peptide, we immunized mice with Lass5-peptide-pulsed and mitomycin-c-treated RMA-S/pUB and RMA-S/B7-1 cells, respectively. PBS-immunization was used as control. Twenty-three-days after immunization, each group was divided into two sub-groups that were challenged with 5×10^6^ cells/mouse of live RMA-S/B7-1 or RMA-S/pUB cells, respectively. Tumor sizes were measured twice a week after challenge with live tumor cells. The tumors appeared in all mice during the initial week in control PBS-immunized groups while the tumors appeared in most mice at 1.5 weeks in tumor-immunized groups (table 2, Fig. 1B-e insert), suggesting that antitumor immunity was established in tumor-immunized groups. This established immunity dramatically inhibited the growth of B7-1 expressing tumors at 1.5 weeks (table 2). During this time point, both RMA-S/pUB- or RMA-S/B7-1-immunized mice challenged with RMA-S/B7-1 cells had tumors that were much smaller in size, and tumors were found in only two out of nine mice, compared to those challenged with the RMA-S/pUB cells in which larger tumors grew quickly in all mice. The difference in tumor sizes between RMA-S/pUB- and RMA-S/B7-1-cell challenged groups at 1.5 week time point was statistically significant (P<0.05). Results suggested that anti-tumor immunity at the effector phase played an important role in inhibiting B7-1 expressing tumor growth. After the initial two weeks of tumor growth, the RMA-S/pUB tumors continued to grow quickly in both RMA-S/pUB and RMA-S/B7-1 immunized mice while no tumors could be detected in the immunized mice challenged with RMA-S/B7-1 cells (Fig. 1B-e and 1C). In PBS-immunized mice, RMA-S/pUB and RMA-S/B7-1 tumors continued to grow dramatically except in one mouse in which the RMA-S/B7-1 tumor had regressed during initial 1.5 weeks (data not shown). Our results suggested that a major component of the anti-B7-1 expressing tumor immunity is T effectors but not NK effectors because: 1) the RMA-S/B7-1 tumors grew quickly in PBS-immunized mice while no RMA-S/B7-1 tumors appeared in tumor-immunized mice at initial week and 2) NK activity could only inhibit less than 1×10^6^ challenged B7-1 expressing RMA-S cells per mouse [22]. In our experiment, 5×10^6^ tumor cells per mouse were injected. To further confirm T effectors provided anti-RMA-S/B7-1 tumor protective immunity, we treated the peptide-pulsed RMA-S/pUB-immunized mice with anti NK1.1 mAb before live cell challenge. Figure 1A (b, c and d) indicated that anti-NK1.1 mAb treatment depleted NK cells in the mice. These mice challenged with RMA-S/pUB or RMA-S/B7-1 cells displayed tumor growth patterns (Fig. 1B-f) similar to the peptide-pulsed RMA-S/pUB-immunized mice without anti-NK1.1 mAb treatment (see Fig. 1B-e insert). The RMA-S/B7-1 cells in the mAb-treated mice grew and formed small tumors that disappeared at week 2 after tumor cell challenge while the RMA-S/pUB cells continuously grew to form large tumors in the mAb-treated mice (Fig. 1B-f). Statistical analysis of tumor sizes indicated significant differences between the two mouse groups during the initial week and 1.5 week time points (P<0.05 and<0.01 respectively). NK activities could play an auxiliary function in controlling RMA-S/B7-1 tumor growth. In the NK depleted and tumor-immunized mice, RMA-S/B7-1 tumors appeared at initial week and disappeared at week 2 (table 2; Fig. 1A-f), while in the tumor-immunized mice RMA-S/B7-1 tumors appeared at 1.5 weeks and disappeared at week 2 (Fig. 1A-e insert). These results indicated that NK activity could only control early or late appearance of RMA-S/B7-1 tumors and could not inhibit tumor growth.

**Table 2:** Tumor formation in the mouse groups during the initial time points.

Mice immunized With or without Tumor cells	Challenge of live tumor cells	
	RMA-S/pUB Number ofmice with tumor	RMA-S/B7-1 Number of mice with tumor
RMA-S/pUB-immunizedgroup	4*	1*
RMA-S/B7-1-immunized group	4*	1*
PBS immunized group	4#	4#
RMA-S/pUB- and mAb treated group	4#	4#

# indicates that tumors appear at initial week after the inoculation.

*indicates that tumors appear at initial 1.5 weeks after the inoculation. Total mice per group were shown in the Material and Method Section.

### Bulk-culture T cells more efficiently kill RMA-S/B7-1 cells, and the killing activities require the B7-1/CD28 axis

**Figure 2:**
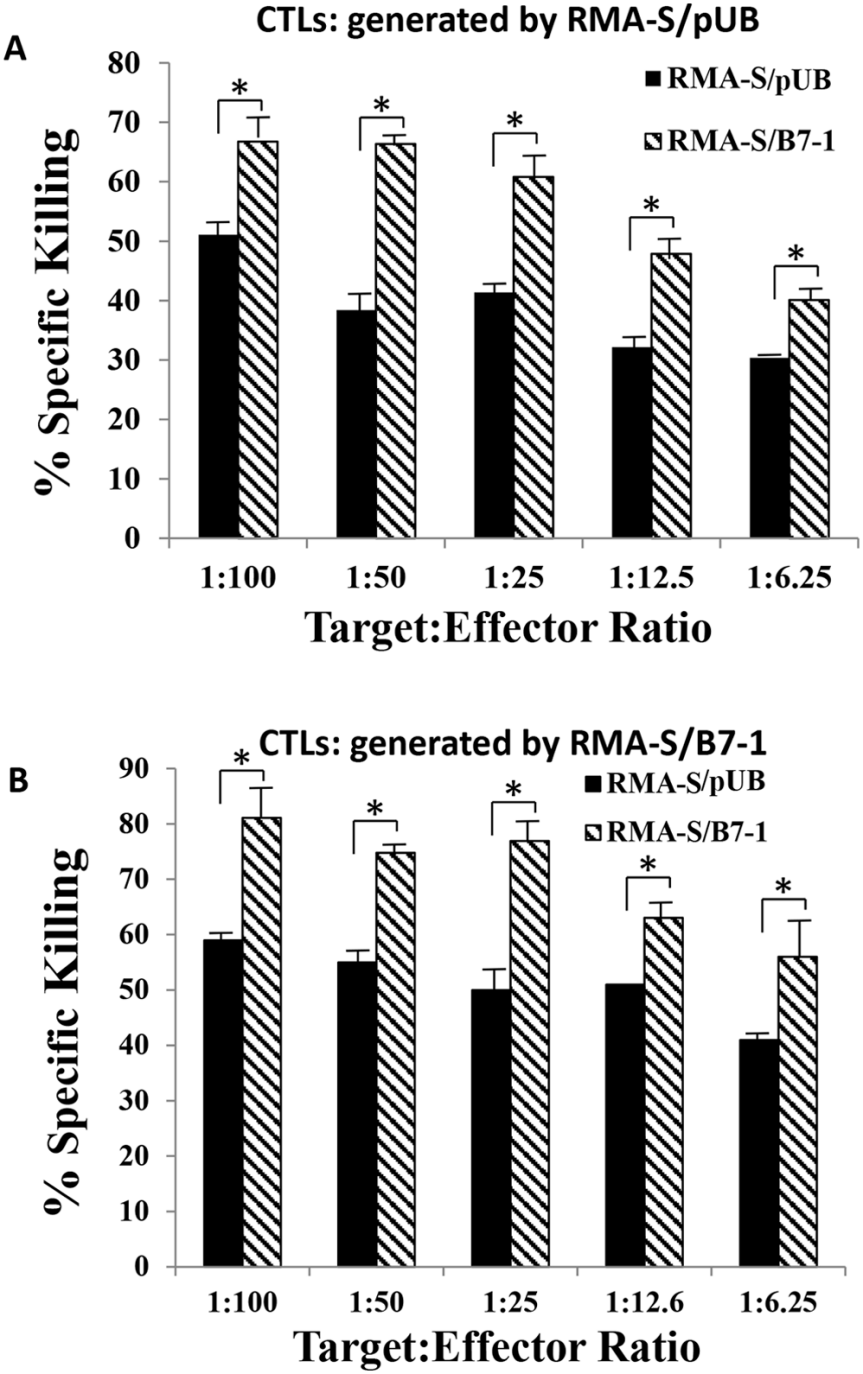
Efficient killing of B7-1 expressing tumor cells by bulk culture T cells. *In vitro* ^51^Cr-release assays were conducted. (A): Bulk-culture T effectors were generated by immunizing mice with Lass5 peptide-pulsed mitomycin-c-treated RMA-S/pUB cells. (B): Bulk-culture T effectors were generated by immunizing mice with Lass5 peptide-pulsed mitomycin-c-treated RMA-S/B7-1 cells. One out of three experiments with similar results was shown. * indicated that P-values were less than 0.05.

To confirm *in vivo* experiments, *in vitro* ^51^Cr-release assays were performed. Two T-cell bulk cultures generated by immunization of mice with Lass5-peptide-pulsed and mitomycin-C-treated RMA-S/pUB or RMA-S/B7-1 cells were used to determine if the B7-1/CD28 axis could enhance T-cell killing activity. Figure 2 showed that two T-cell bulk cultures killed B7-1-expressing RMA-S/B7-1 targets more efficiently than RMA-S/pUB targets (Fig. 2A and B). These results suggested that the role of B7-1 molecule in increasing immune response at the effector phase could occur in Lass5-peptide-stimulated T-cell bulk cultures.

**Figure 3:**
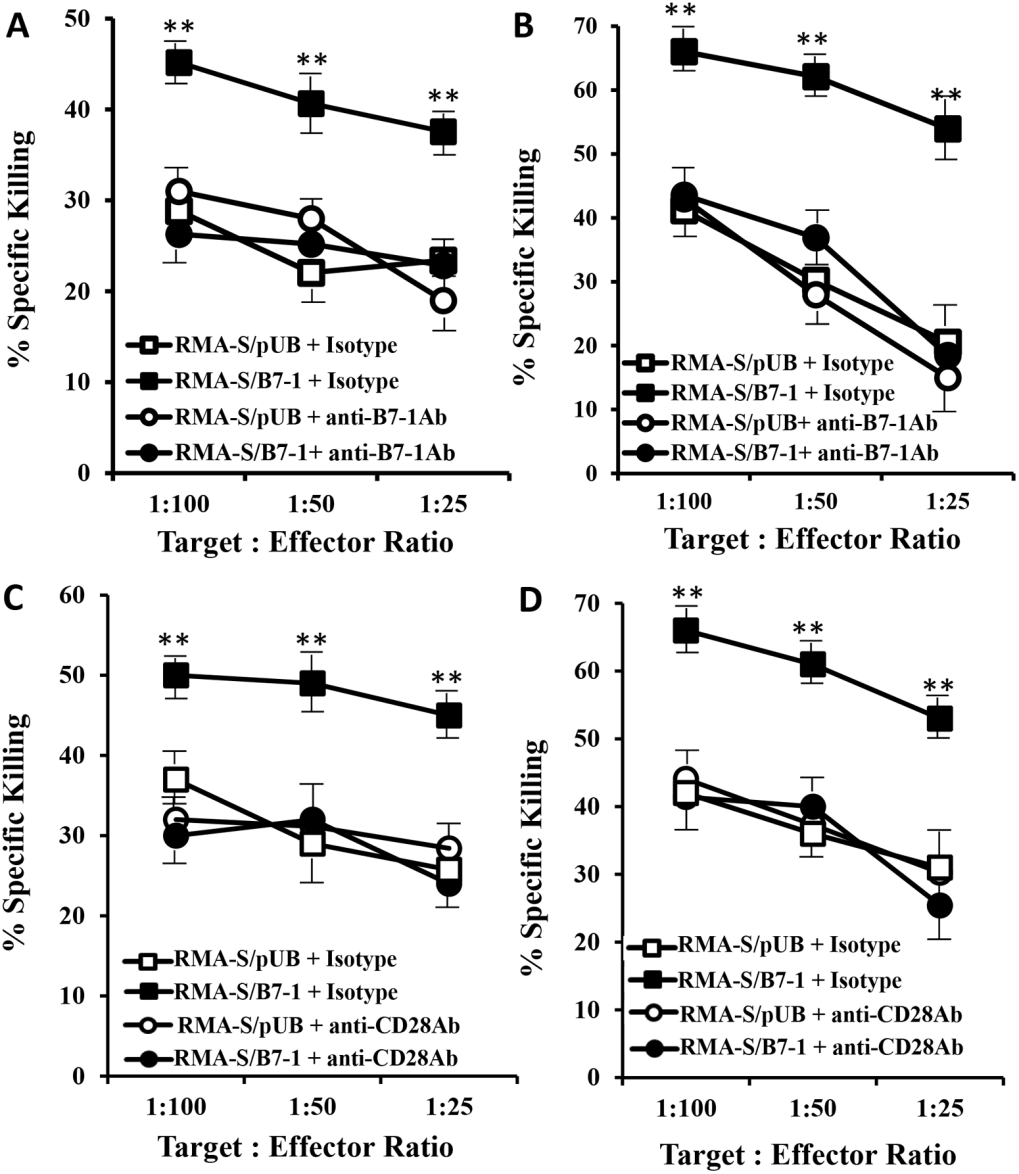
Effects of anti-CD80 and CD28 antibodies on reducing killing activities of bulk culture T effectors against RMA-S/B7-1 cells. Lift-panel (A and C): The cytolytic T effectors were generated by immunization of mice with mitomycin-c-treated RMA-S/pUB cells pulsed with Lass5 peptide. Right-panel (B and D): The cytolytic T effectors were generated by immunization of mice with mitomycin-c-treated RMA-S/B7-1 cells pulsed with Lass5 peptide. Up-panel (A and B):^ 51^Cr-labeled RMA-S/B7-1 and RMA-S/pUB target cells were incubated with either anti-mouse B7-1 mAb or relevant IgG-control. After incubation, the cells were then incubated with antigen-specific bulk culture T effectors for *in vitro* ^51^Cr-release assays. Bottom-panel (C and D): Cytolytic bulk culture T effectors were incubated with either anti-mouse CD28 mAb or relevant IgG-control. After incubation, the T-cells were then incubated with ^51^Cr-labeled RMA-S/B7-1 and RMA-S/pUB target cells for *in vitro* ^51^Cr-release assays. ** indicated that P-values were less than 0.05 among ‘RMA-S/B7-1+ Isotype’ and other targets at each ‘Target: Effector’ ratio.

To confirm enhanced T-cell killing activity was associated with the B7-1/CD28 axis, blocking antibodies against B7-1 and CD28 molecules were used. We first performed assays to block the B7-1/CD28 axis using a mAb against mouse B7-1, and an IgG isotype antibody was used as a control. After incubation of RMA-S/B7-1 targets with the mAb or the isotype antibody at room temperature for 1 hour, the targets were mixed with effectors, and the effector killing activities were determined. Results showed that T-cell killing activities against the antibody-incubated RMA-S/B7-1 targets were reduced to a level similar to those observed in RMA-S/pUB cells incubated with isotype-control antibody while isotype-blocked RMA-S/B7-1 cell killing remained at higher levels (Fig. 3A and B). In addition, blocking of the B7-1/CD28 axis by using a mAb against mouse CD28 displayed similar results (Fig. 3C and D). These assays suggested that enhanced killing activities of T effectors required B7-1/CD28 binding.

It has been reported that NK activity can be triggered *in vitro* by B7-1, and this occurred even in the absence of CD28 and could not be blocked by anti-CD28 mAb [23]. Our preparation of T-cell bulk-cultures displayed killing activities for RMA-S/B7-1 targets being reduced by anti-CD28 mAb, suggesting that the role of NK cells was negligible.

### B7-1/CD28 axis plays a major role in increasing LnB5 T-cell activation

**Figure 4:**
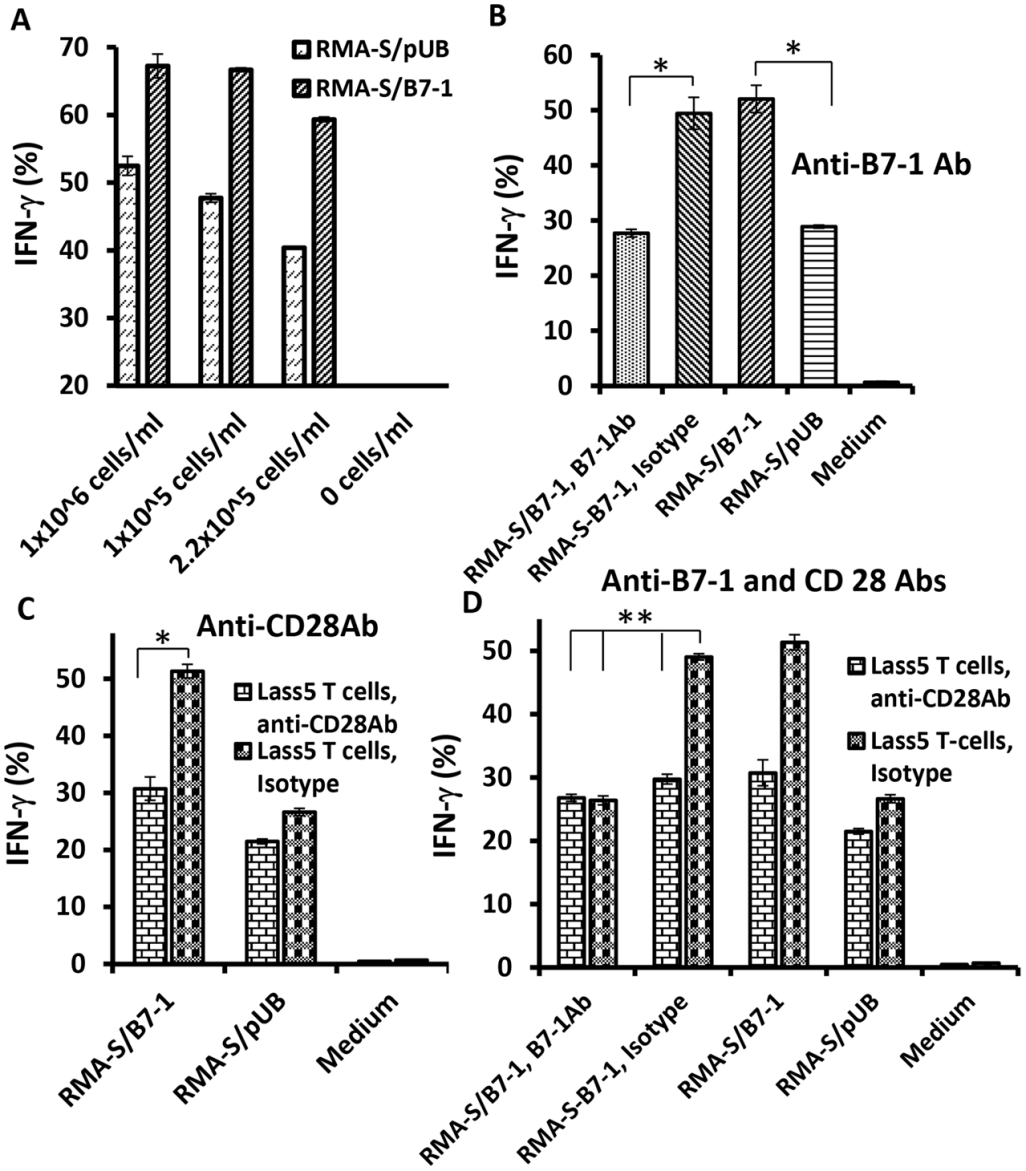
Importance of B7-1:CD28 axis in enhancing a Lass5 specific LnB5 T-cell clone activation. The RMA-S/pUB and RMA-S/B7-1 transfectants were used as targets recognized by a Lass5 specific LnB5 T-cell clone. Lass5 specific T-cell clone activation detected by the intracellular IFN-gamma release assays were conducted with stimulators RMA-S/pUB and RMA-S/B7-1 cells in (A) to (D). (A): 8×10^3^ T-cells were incubated with indicated amounts of RMA-S/pUB and RMA-S/B7-1 cells. (B): 8×10^3^ T-cells were incubated with 1×10^5^ stimulators that previously incubated with either anti-B7-1 mAb or isotype control (for RMA-S/B7-1). (C): 8×10^3^ T cells were incubated with either anti-CD28 mAb or isotype control before co-culture with 1×10^5^ stimulators (RMA-S/pUB or RMA-S/B7-1). (D): Before co-culture of the T-cells and stimulators, 8×10^3^ T-cells were incubated with either anti-CD28 mAb or Isotype control and 1×10^5^ RMA-S/B7-1 stimulator cells were incubated with either anti-B7-1 mAb or Isotype control. One out of at least two experiments with similar results was shown. * and ** indicated that P-values were less than 0.05.

To confirm that the role of the B7-1/CD28 axis in delivering a signal into and activating the T-cells at the effector phase was not due simply to binding, the LnB5 T-cell clone specific for the Lass5 peptide [13] was employed. We incubated the LnB5 cells with different amount of either RMA-S/B7-1 or RMA-S/pUB cells and measured the concentration of IFN-gamma secretion by the LnB5 T-cells. Results clearly showed that RMA-S/B7-1 cells stimulated T-cell activation more efficiently than the RMA-S/pUB cells as indicated by more IFN-gamma secretion (Fig. 4A). Enhanced T-cell activation was confirmed to be due to the B7-1/CD28 axis because blocking B7-1/CD28 binding between RMA-S/B7-1 targets and LnB5 effectors by either anti-B7-1 or anti-CD28 antibodies or both reduced IFN-gamma secretion to the levels similar to that of LnB5 T-cells incubated with RMA-S/pUB cells (Fig. 4B, C and D). These results indicate that the B7-1/CD28 axis provides a second signal, triggering enhancement of Lass5 antigen specific T-cell activation at the effector phase.

### Requirement of B7-1/CD28 signaling at the effector phase of immunity is overcome by Lass5-overexpressing targets

Why does enhanced response to Lass5 antigen require the secondary signal at the effector phase? The possible reasons are 1) the Lass5 peptide has a low affinity for H-2D^b^ binding and/or 2) the Lass5 peptide is generated at a limited level. Both of these possibilities would reduce antigenic peptide surface stability or expression. These situations may reduce the strength of the first signal and therefore require help by the secondary signal to efficiently activate function of T effectors. We have previously performed peptide-binding and peptide-stability assays demonstrating binding and stability of the Lass5 peptide to H-2D^b^ at levels comparable to the levels of high affinity binders such as the viral gp33 epitope (KAVYNFATM) from LCMV [14]. Computer modeling analysis of Lass5 peptide and two immunodominant viral epitopes, ASNENMETM from the influenza-A virus and KAVYNFATM from LCMV virus, demonstrated that the relative binding capacity of the Lass5 peptide is weaker than influenza-A viral peptide but stronger than LCMV viral peptide (data not shown). These results suggested that binding capacity of the Lass5 epitope to the H-2D^b^ molecule is similar to immunodominant viral epitopes.

To test if increased Lass5 expression could overcome the requirement of the B7-1/CD28 axis for enhancing immune response, RMA-S/B7-1 and RMA-S/puB cells were further transfected with a Lass5 (Trh4) cDNA-carrying LZRS retroviral vector. Lass5 mRNA over-expression in the transfectants was detected by quantitative PCR (no antibody available). Long and short Lass5 transcripts were detected, and only the long transcript contained a Lass5 coding sequence [13]. Table 3 shows that both RMA-S/B7-1.Trh4 and RMA-S/pUB.Trh4 cells expressed higher levels of Lass5 mRNA compared to that detected in RMA-S cells. The levels of the increased Lass5 transcripts in RMA-S/B7-1.Trh4 and RMA-S/pUB.Trh4 cells were about 822 and 535 respectively.

**Table 3:** Lass5 mRNA expression in RMA-S transfectants.

Lass5	RMA-S		RMA-S/pUB.Trh4		RMA-S/B7-1.Trh4	
mRNA	Mean	StDev	Mean	StDev	Mean	StDev
Long	1.00	0.12	534.84	26.09	821.84	33.01
Short	3.86	0.32	12.21	1.25	8.27	1.19

Note: Lass5 mRNA expression was determined by quantitative PCR using specific primers. Levels of Lass5 mRNA expression of two natural splice variants (long and short) were normalized with mRNA of the GAPDH housekeeping gene. Only long transcript is coding for the Lass5 peptide MCLRMTAVM.

**Figure 5:**
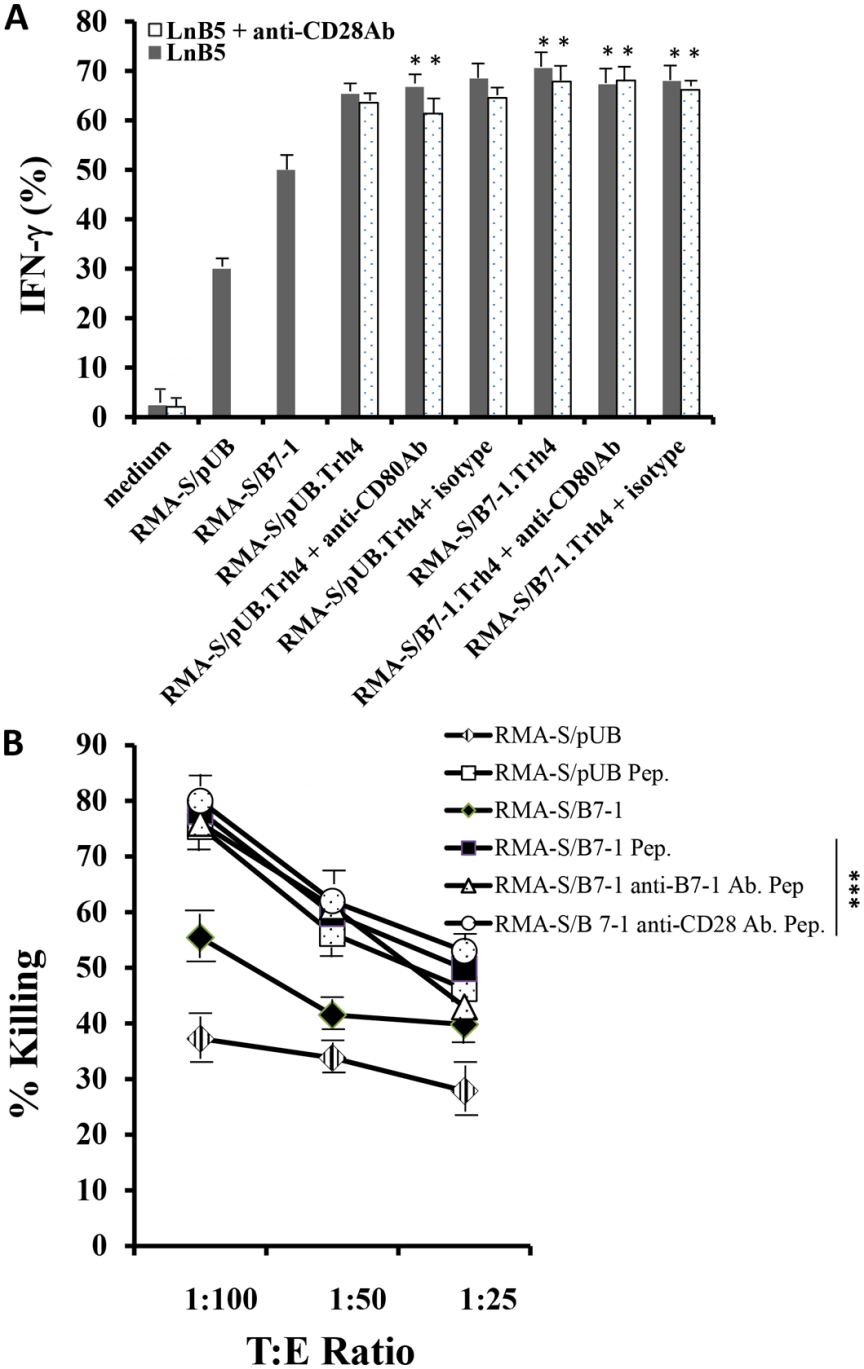
Increase in Lass5 expression Bypasses B7-1/CD28 requirement for T effectors’ response. Lass5 specific LnB5 T-cell clone (A) and T-cell bulk culture (B) were used to determine B7-1/CD28 requirement. (A): Lass5 high expressing RMA-S/pUB.Trh4 and RMA-S/B7-1.Trh4 cells were used as targets that were recognized by LnB5 T-cell clone. The antibodies against CD80 (B7-1) or CD28 molecules were used to block B7-1/CD28 axis. The isotype Ig was used as a control. (B): Lass5-peptide (50 micromole) pulsed RMA-S/pUB and RMA-S/B7-1 cells were used as targets that were recognized by T-cell bulk culture for ^51^Cr-release assays. Pep means Lass5 peptide. One out of two experiments with similar results for each assay was shown. * * and *** indicated no statistical significance.

Overexpression of Lass5 mRNA in transfectants enhanced LnB5 T-cell recognition. Both RMA-S/B7-1.Trh4 and RMA-S/pUB.Trh4 cells stimulated LnB5 effectors to secrete IFN-gamma at levels higher than that found in Trh4-untransfected counterparts (Fig. 5A), suggesting that higher IFN-gamma secretion in the T-effectors was induced by the recognition of increased number of D^b^/Lass5 complexes on the surface of the transfectants. In addition, LnB5 T-effectors stimulated by RMA-S/B7-1.Trh4 or RMA-S/pUB.Trh4 cells secreted similar levels of IFN-gamma (Fig. 5A). Apparently, B7-1 expression on the RMA-S/B7-1.Trh4 cells provided a negligible role in serving as a secondary signal for T-cell activation. This was further confirmed by antibody blocking assays in which both anti-B7-1 and/or anti-CD28 antibodies could not reduce T-effector activation (Fig. 5A). The results might indicate that the transfectants expressed an increased number of D^b^/Lass5 complexes which provided a stronger first signal for T effector activation and thus overcame the requirement for the B7-1/CD28 signal. To further confirm the increased number of D^b^/Lass5 complexes being a critical factor for providing enhanced T-cell killing activity that bypass the requirement of B7-1/CD28 signaling, RMA-S/B7-1 and RMA-S/pUB cells were pulsed with Lass5 peptide as targets in polyclonal T-cell based ^51^Cr-release assays. The peptide-pulsed targets should express much more surface D^b^/Lass5 complexes, and they displayed higher responses for T-cell killing, compared to RMA-S/B7-1 and RMA-S/pUB cells (Fig. 5B). The blockage of the B7-1/CD28 axis by the antibodies did not reduce T-cell killing activities on the peptide-pulsed RNA-S/B7-1 targets (Fig. 5B).

Taken together, the results indicated that naturally expressed Lass5 epitope provides a relatively weak first signal for T-effector response and thus the secondary signal is required. Increasing the number of Lass5 epitopes on the cell surface compensates for the inadequate first signal and bypasses the requirement for the B7-1/CD28 secondary signal for T-effector responses.

## Discussion

We have demonstrated that, in comparison with RMA-S/pUB cells, RMA-S/B7-1 cells are more efficiently recognized by Lass5 specific T-cell clones or bulk-cultures of T effectors. The enhanced T-cell based immune response against RMA-S/B7-1 cells occurs at the effector phase of the immunity and requires binding of B7-1 on tumor cells to CD28 on antigen specific T effectors. This requirement can be overcome by an increase in Lass5 expression in tumor cells.

In antitumor immunity, B7-1-transfected tumor cells are potent immunogens which provoke robust T-cell-based antitumor immune reactions [10], [11]. The existence of the enhanced immunity may reflect the involvement of tumor-direct priming for antitumor-specific T-cell generation [12]. Although numerous accumulated data support the importance of B7-1 in the induction phase of antiviral and antitumor immunity, the involvement of this molecule in the effector phase has emerged recently. There is a report indicating that in influenza-infected mice, B7-expressing dendritic cells (DCs) trigger both CTL cytotoxicity and release of inflammatory mediators while B7-negative epithelial cells trigger only CTL cytotoxicity [24]. Furthermore, the authors show that inhibiting B7/CD28 interactions significantly decreases the release of inflammatory mediators and that this decrease coincides with a corresponding reduction in mediator-producing CD8^+^ T cells [24]. Another report indicates that absence of costimulation by B7/CD28 association at the effector phase leads to reduced survival of influenza virus specific effector cells [25]. Apparently, B7/CD28 association at the effector phase was associated with an increase in the number of virus specific CD8^+^ T cells. In antitumor immunity, one report suggested that B7-1 was involved in enhanced antitumor immunity at the effector phase. Bai et al [26], by determining the sizes of murine B7-1 positive and negative tumors in tumor-carrying RAG−/− mice that were administered tumor-antigen specific CTLs, found that the CTLs inhibited growth of the B7-1 positive tumors more efficiently than the B7-1 negative counterparts. These results are very similar to our *in vivo* results (Fig. 1B-e insert). Our work *in vitro* expands upon these *in vivo* findings by removing confounding factors *in viv*o to further confirm that B7-1/CD28 signaling is involved at the effector phase of antitumor immunity. Specifically, our results of CTL activation and killing assays provide important information that directly indicates the association of B7/CD28 signaling with the effector phase of antitumor immunity because our *in vitro* working system contains only cloned or bulk-cultured CTLs with B7-1 positive or negative targets and thus this system eliminates possible confounding factors. Our results from *in vitro* experiments also indicate that the same number of CTLs provide higher activation/killing activities against B7-1 positive than B7-1 negative tumor cells. This differs from that reported by other research groups [24], [25] who demonstrated that the influenza viral specific immune responses at the effector phase with or without B7/CD28 association were influenced by the numbers of the CTLs. Of particular note, the enhanced CTL activities in our experiments cannot be attributed simply to B7-1/CD28 association leading to target/T-cell close binding, because the association activates the T effectors to secrete more IFN-gamma suggesting that a signal is delivered into the T effectors (Fig. 4).

Others have demonstrated that NK activities were involved in B7-1 expressing RMA-S cells *in vitro* and *in vivo* [22], [23]. In *in vitro* assays, the report [23] indicated that NK activities were independent of B7-1/CD28 association, since an anti-CD28 mAb was unable to block NK reactivity. In our experiments, the enhanced activity of the polyclonal T effectors can be blocked by an anti-CD28 mAb (Fig. 3C and D), suggesting negligible NK activities in the T-cell bulk-cultures. In *in vivo* assays, NK activities were reported [22] to control B7-1 expressing RMA-S tumor growth, and this control was dependent on initial cell numbers in the inoculate. In the case of inoculation with more than 1×10^6^ B7-1 expressing tumor cells per mouse, NK activities only temporally inhibited but did not block tumor formation and growth [22]. Our results support this point of view (Fig. 1B-e insert). In PBS-immunized mice, all RMA-S/B7-1-inoculated mice grew tumors during the first week and the growth rate of the tumors was decreased 2.34-fold, compared to growth rate of the RMA-S/pUB tumors. However, both B7-1 positive and negative tumors grew quickly in the following weeks with one exception in which one RMA-S/B7-1 tumor was regressed.

T-cell-based immunity but not NK activity plays a major role in controlling B7-1 expressing RMA-S tumor growth at the effector phase. Our *in vivo* tumor immunization and NK depletion experiment (Fig. 1B-f) demonstrates this issue. Without NK activity, antigen specific T effectors inhibited growth of B7-1 positive RMA-S tumors more efficiently than growth of B7-1 negative counterparts. At least, the results at the initial week reflect inhibitive function of T effectors at the effector phase. The following weeks may suggest both the induction and effector phase of T cell immunity being activated by challenged B7-1 positive tumor stimulation.

Lass5 peptide is a suitable H-2D^b^ binder, similar to immunodominant viral epitopes [14] (and unpublished data). Its expression at a limited level on the surface of RMA-S cells was suggested by the evidence indicating that it cannot be presented by TAP-proficient RMA cells [13] (because of other TAP-dependent peptides’ competition) and can be presented by Lass5-transfected RMA cells [14]. Transfection of Trh4 (Lass5) gene into or Lass5 peptide-pulse on RMA-S/B7-1 and RMA-S/pUB cells enhances T-cell responsiveness and bypasses the requirement for B7-1/CD28 signaling at the effector phase (Fig. 5A and B). Reports showed that the association between MHC-I/peptide complexes on targets and T-cell receptors (TCRs) on T cells served as first signal for T-cell responsiveness and this signal requires clustering of the TCRs with the MHC-I/peptide complexes at the interface [27]–[29]. Recent report indicated that the density of the MHC-I/peptide complexes can regulate TCR signaling [30]. Our results indicating enhanced T-cell responsiveness and decreased B7-1/CD28 requirement (Fig. 5A and B) may be ascribed to increased Lass5 peptide densities on target cells associated with relative larger TCR clusters on the effectors that provide a stronger first signal for T-cell responses without requirement of B7-1/CD28 signaling.

Besides B7-1/CD28 signaling, association of B7-1 with cytotoxic T lymphocyte-associated antigen 4 (CTLA-4) provides another signal to T-cells. This B7-1/CTLA-4 signal, unlike the B7-1/CD28 signal, terminates T effector activation [31]. Blocking B7-1/CD28 association by anti-CD28 mAb reduced T effector activation and killing activity (Fig. 3C–D and 4C–D). The reduction in T-effector function cannot be attributed to blockage of B7-1/CD28 positive signal thereby activating the B7-1/CTLA-4 negative signal, because blocking both signals by combinations of anti-CD28 and anti-CTLA-4 (clone: 9H10) mAbs did not recover T-effector killing activity against RMA-S/B7-1 targets (data not shown). In Trh4-transfected or Lass5 peptide-pulsed RMA-S/B7-1 target system (Fig. 5), blockage of B7-1/CD28 association by anti-CD28 mAb did not activate the B7-1/CTLA-4 negative signaling because reduction of T-effector activities was not observed. It is not clear that why CTLA-4 does not promote a negative signal to inhibit T-effector function in our working system. Some reports have provided an opposite evidence in which CTLA-4 played active signal for T-cell activation [5], [32]. In our current work, the results of B7-1/CTLA-4 signaling are limited but we are interested in investigating further.

TAP2-deficient RMA-S cells can present many different TAP-independent antigens, as demonstrated by different T-cell clones being generated [13]. In future studies, we will investigate if the results observed with Lass5 antigenic peptide presentation can be expanded to other TAP-independent antigens. If these antigens display similar results, it suggests that 1) T-cell responses to TAP-independent antigens require B7-1/CD28 signaling at the effector phase and 2) a potential mechanism in which the first signal strength regulates the requirement of secondary B7-1/CD28 signaling shown in Lass5 antigen presentation can be confirmed to be an important role for T-cell response to TAP-independent antigens at the effector phase. Since many types of human cancers down-regulate TAP molecules [33], [34], understanding how T-cells respond to these types of cancers may provide useful information for cancer immunotherapy.
